# Yeast *β*-glucan supplementation lowers insulin resistance without altering microbiota composition compared with placebo in subjects with type II diabetes: a phase I exploratory study

**DOI:** 10.1017/S0007114524002526

**Published:** 2024-11-14

**Authors:** Peter Cronin, Cian Hurley, Andrew Ryan, María Zamora-Úbeda, Ashokkumar Govindan, Catherine Stanton, Ger P. Lane, Susan A. Joyce, Paul W. O’Toole, Eibhlís M. O’Connor

**Affiliations:** 1 Department of Biological Science, University of Limerick, Limerick, Republic of Ireland; 2 APC Microbiome Ireland, University College Cork, Cork, Republic of Ireland; 3 School of Microbiology, University College Cork, Cork, Republic of Ireland; 4 School of Medicine, University of Limerick, Limerick, Republic of Ireland; 5 Department of Anatomy and Neuroscience, University College Cork, Cork, Republic of Ireland; 6 Teagsac Food Research Centre, Moorepark, Fermoy, Cork, Republic of Ireland; 7 School of Biochemistry and Cell Biology, University College Cork, Cork, Republic of Ireland; 8 Health Research Institute, University of Limerick, Limerick, Republic of Ireland

**Keywords:** Type II diabetes mellitus, Yeast *β*-glucan, Glucose control, Bile acids

## Abstract

The increased global prevalence of type II diabetes mellitus (T2DM) is associated with consumption of low fibre ‘Western diets’. Characteristic metabolic parameters of these individuals include insulin resistance, high fasting and postprandial glucose, as well as low-grade systemic inflammation. Gut microbiota composition is altered significantly in these cohorts suggesting a causative link between diet, microbiota and disease. Dietary fibre consumption has been shown to alleviate these changes and improve glucose parameters in individuals with metabolic disease. We previously reported that yeast *β*-glucan (yeast beta-1,3/1,6-D-glucan; Wellmune) supplementation ameliorated hyperinsulinaemia and insulin resistance in a murine model. Here, we conducted a randomised, placebo-controlled, two-armed dietary fibre phase I exploratory intervention study in patients with T2DM. The primary outcome measure was alteration to microbiota composition, while the secondary outcome measures included markers of glycaemic control, inflammation as well as metabolomics. Patients were supplemented with 2·5g/day of maltodextrin (placebo) or yeast *β*-1,3/1,6-D-glucan (treatment). Yeast *β*-glucan (Wellmune) lowered insulin resistance compared with the placebo maltodextrin after 8 weeks of consumption. TNF*α* was significantly lower after 4 weeks of *β*-glucan supplementation. Significantly higher fecal concentrations of several bile acids were detected in the treatment group when compared with the placebo after 8 weeks. These included tauroursodeoxycholic acid, which was previously shown to improve glucose control and lower insulin resistance. Interestingly, the hypoglycaemic and anti-inflammatory effect of yeast *β*-glucan was independent of any changes in fecal microbiota composition or short-chain fatty acid levels. Our findings highlight the potential of yeast *β*-glucan to lower insulin resistance in patients with T2DM.

The prevalence of Type II diabetes mellitus (T2DM) continues to rise internationally, particularly in industrialised nations^(1)^. There has been a major increase in cases reported over the last few decades. Estimates in 1980 reported 108 million cases while in 2014 approximately 422 million cases were reported worldwide^(2)^. More recently, the International Diabetes Federation has estimated that this number is now 537 million and is expected to continue to rise to 643 million by 2030 and 783 million by 2045^(3)^. T2DM is characterised by low-grade systemic inflammation and dysfunctional glucose and lipid metabolism^(4)^. T2DM pathogenesis typically sees a steady decline in pancreatic *β*-cell function resulting in lower levels of insulin secretion which is concurrent with an increase in insulin resistance in adipocytes, hepatocytes and skeletal muscle^(5)^. Several studies have reported that gut microbiota composition is significantly altered in patients with T2DM when compared with controls^(6–8)^. The drug metformin is the most prescribed pharmacotherapy and the first-line treatment for T2DM^(9)^. The exact mechanisms through which metformin alleviates hyperglycaemia and insulin resistance are not fully understood. However, it is accepted that metformin can reduce glucose production in the liver through the activation of both AMP-activated protein kinase dependent^(10–12)^ and AMP-activated protein kinase independent pathways^(13,14)^ while also blocking reabsorption of glucose in the kidneys. It has also been shown that metformin can exert its actions through pathways in the gut^(15,16)^. Metformin was shown to significantly alter gut microbiota composition and increase the availability of the health promoting short-chain fatty acids (SCFA), which are in part thought to mediate its hypoglycaemic effect on the host^(15)^.

Increased incidence of T2DM in industrialised nations directly correlates with increased consumption of the calorie-dense Western diet, which is typically low in dietary fibre and high in sugars and saturated fat^(17–19)^. Dietary fibre can exert a beneficial effect on host physiology by modulating gut microbiota composition and influencing both metabolism and inflammation^(20)^. Certain species of colonic microbiota can ferment dietary fibre to produce SCFA which in turn lower insulin resistance though multiple mechanisms^(21–23)^. SCFA can also act as histone deacetylase inhibitors. Histone deacetylase inhibition can suppress pro-inflammatory macrophage responses, regulate cytokine expression in T cells and generate regulatory T cells thus exerting an anti-inflammatory effect^(24,25)^. Although metformin is an effective first-line treatment for T2DM, dietary fibre intervention is now being recognised as an effective approach to help mitigate metabolic and inflammatory disease. Zhao *et al.* (2018) found that a diet high in dietary fibre promoted the growth of a number fibre fermenting microbiota (SCFA-producing), which coincided with significant reductions of fasting glucose and HbA1c in patients with T2DM^(26)^.

*β*-glucans are a type of dietary fibre composed of D-glucose monomers that are connected by *β*-glycosidic bonds and are naturally found in various organisms such as plants, algae, fungi and bacteria^(27)^. Importantly, *β*-glucans have different functions depending on their structural composition. Specifically, *β*-glucans have been shown to modulate immune function^(27–29)^, lower cholesterol levels^(30)^ as well as regulating glucose^(31)^ and lipid metabolism^(32)^. Previously, a pre-clinical study, where male C57BL/6J mice were fed a high-fat diet, inoculated with microbiota from obese T2DM patients and supplemented with yeast *β*-1,3/1,6-D-glucan (Wellmune), reported that yeast *β*-glucan significantly lowered hepatic lipid production that coincided with higher levels of health-associated microbiota^(33)^. To date, nobody has examined the effect of yeast *β*-glucan (Wellmune) on metabolic health and microbiota composition in humans with T2DM. Thus, the aim of the current study was to test whether yeast *β*-glucan dietary supplementation could alter metabolic and inflammatory phenotype in patients diagnosed with T2DM through changes to fecal microbiota composition and function.

## Materials and methods

### Study design

**Figure 1.**
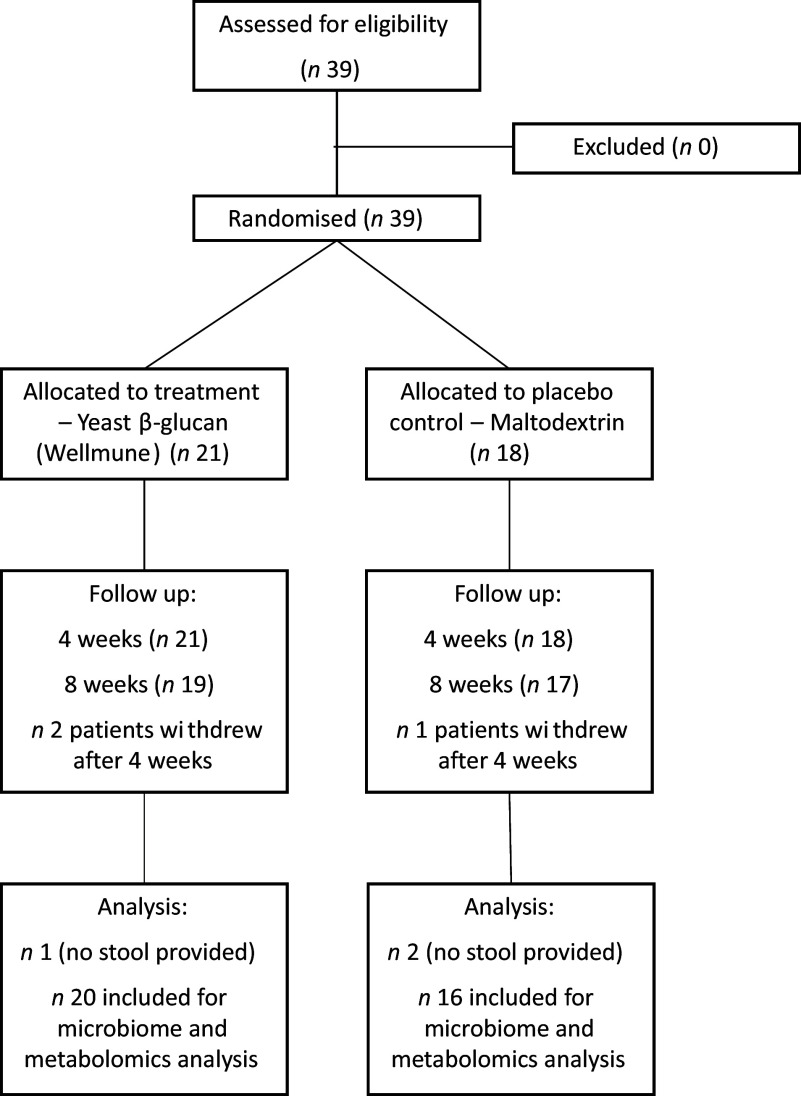
Intervention design. We performed a randomised, placebo controlled, two-arm dietary fibre (yeast *β*-glucan) intervention in patients with T2DM. T2DM, type II diabetes mellitus.

This study was designed as a randomised, placebo-controlled, two-arm phase I exploratory intervention (Fig. 1). The primary outcome measure was alterations to microbiota composition, while secondary outcome measures included markers of glycaemic control, inflammation and metabolomics (fatty acids and bile acids). A power calculation was carried out based on a previous microbiome study^(34)^, using a treatment effect of 25 % change in microbiome beta diversity, we calculated using a delta figure of 17·5 and a power of 0·9 that 20 subjects would allow us detect the minimum difference in phylogenetic diversity, thus achieving statistical significance for the primary outcome measure. Both male and female participants with a previous diagnosis of T2DM, aged between 18 and 85 years, were recruited from the Limerick area between June 2022 and December 2022. At baseline, the majority of patients recruited had abnormal glucose control (defined as fasting blood glucose > 6·1 mmol/l or HbA1C > 42 mmol/mol). Baseline measures of glycaemic control were unavailable for four patients (online Supplementary File 1). Participants who were eligible signed written consent prior to the start of the intervention. Patients enrolled were block randomised (using sequencing numbered products) to take 2·5 g/d of yeast *β*-1,3/1,6-D-glucan (Wellmune) or 2·5 g/d of a placebo maltodextrin for 8 weeks (Fig. 1). Reminders to consume the product were sent to the participants once per week. Yeast *β*-1,3/1,6-D-glucan (Wellmune) was supplied by Kerry, while the maltodextrin placebo was supplied by Bulk. Yeast *β*-glucan was administered in 250 mg capsules of which participants consumed 10 per day (2·5 g total). Capsules could be consumed anytime each day and did not have to be consumed in one go. Maltodextrin placebo was administered in sachets, and participants were instructed to consume with water. To date, several studies investigating the effect of yeast *β*-glucan in humans used a dose of 250 mg/d^(35–38)^. Previously, pre-clinical murine models showed that yeast *β*-glucan (Wellmune) altered fecal microbiota composition, exerted a hypoglycaemic effect and improved insulin resistance using a dose of 50 mg/kg^(39,40)^. While the current study used a significantly higher dose (2·5 g/d) of yeast *β*-glucan than previous human interventions (250 mg/d), the dose was extrapolated from previous pre-clinical studies (50 mg/kg) using the dose by the factor method^(41)^. An equal dose of maltodextrin was selected as a placebo control for this study, a widely accepted placebo used in both T2DM and microbiota interventions^(42–46)^. However, it is important to note that both treatment and placebo products could not be obtained in identical forms and thus blinding was not possible. The study comprised three study visits (baseline, 4 week and 8 week visit) over an 8-week period. All participants were asked to refrain from using other probiotic or nutrition supplements during the course of the intervention and to keep records of dietary intake and physical activity as consistent as possible for the duration of the study. Patients were excluded from the intervention if they had taken antibiotics in the 4 weeks prior to the beginning of the study, if they had a history of alcoholism or gastrointestinal disease, if they adhered to a restricted diet, if they used anticoagulants (e.g. warfarin) or if they had participated in another diet or drug intervention in the preceding three months. All study visits took place at one of two general practices in the greater Limerick city area. Patients were asked, on a weekly basis, about tolerance and gastrointestinal abnormalities in response to administration of either yeast *β*-glucan or maltodextrin. However, no side effects were reported during the study. The study was conducted in accordance with the ICH Guidelines on Good Clinical Practice, and the declaration of Helsinki and approved by the Research Ethics Committee, University of Limerick Hospitals Group, Limerick.

### Data collection

Dietary data were collected at each study visit using a validated FFQ as previously reported^(34)^. Physical activity levels were determined using the International Physical Activity Questionnaire^(47)^. Anthropometric measurements including weight and BMI were determined at each study visit. Changes in bowel habit and stool consistency were also recorded for each patient at every study visit. Habitual dietary fibre intake was measured using a validated questionnaire^(48)^.

### Sample collection

Fresh stool samples were collected from enrolled patients, as close to the study visit as possible using polypropylene screw cap tubes (Sarstedt). The time elapse between sample collection by the participant and freezing at the laboratory varied between 2 and 6 hours. During this period, the samples were kept in the participant’s refrigerator and transported to the study site using a cool pack. Once at the study site, the sample was placed in the refrigerator until processing. Each participant provided a serum sample which was also refrigerated until transportation to the laboratory. Upon arrival at the laboratory, each sample was aliquoted and stored at –80°C for subsequent analysis.

### DNA extraction and shotgun metagenomics

DNA was extracted from fecal samples using the repeated bead-beating method as previously described^(49)^. Paired end shotgun metagenomic sequencing was performed at Novogene (Cambridge, UK) on an Illumina NovaSeq platform using 2 × 150 bp chemistry.

### Processing of microbiome sequencing data

Shotgun metagenomic raw reads were first subject quality filtering using the default settings of Trimmomatic software (version 0.39)^(50)^. Reads were subsequently mapped to the human genome (hg37) using Bowtie2^(51)^ and removed from the dataset in order to control for host contamination. The taxonomy of each read was determined using MetaPhlAn4 (version 4.05)^(52)^. Furthermore, each read was functionally annotated using the HUMAnN3 package (version 3.6)^(53)^.

### Metabolomics sample preparation

For extraction of bile acids and fatty acids from each fecal samples, 100 mg of fecal material was added individually into sterile screw capped tubes pre-filled with 1·4 mm diameter ceramic beads (Roche Diagnostics, Switzerland) to facilitate extraction. Samples were spiked with deuterated (D) internal standards (D4 cholic acid, D4 chenodeoxycholic acid, D4 deoxycholic acid and Steraloids) and ice-cold 50 % methanol was added to each. Samples were then subjected to three, 30 second rounds of beating (MagNA Lyser Instrument (RocheTM) at 6000 rpm before centrifugation at 10 000 × ***g*** for 10 min. Extracted mixtures were dried before metabolite extraction in ice-cold 95 % acetonitrile containing 5 % formic acid. The dried extracts were reconstituted in 150 μl of 50 % methanol, transferred into glass vials (Waters Ltd) and then applied to ultra-performance LC-MS (UPLC-MS). Sample preparation for SCFA quantification was carried out as previously described^(54,55)^. Briefly, we placed 200–400 mg of fecal material into a sterile 2 ml microcentrifuge tube and added sterile PBS. Samples were vortexed for 5 min and centrifuged at 16000 ***g*** for 30 min. The resulting supernatant was taken into a 2 ml microfuge tube and centrifuged again at 16 000 g for 30 min. Samples were then subject to two filtration steps, using ultrafree-MC-SV 5·0 µm and 0·2 µm filters centrifugal filters.

### Metabolomics analysis

UPLC tandem mass spectrometry experiments were performed with samples injected in triplicate in accordance with^(56)^. Briefly, 5 µl of extracted bile acid were injected onto a 50 mm Acquity UPLC BEH C18 column (Waters Corp.) and were eluted using a 25-min gradient of 42 % A to 68 % B (A, water pH 4, 7·5 mM NH_4_ acetonitrile.; B, 95 %MeOH:5 %acetonitrile pH 4) at a flow rate of 300 μl/min and column temperature of 45°C. Samples were analysed using XEVO-G2QTOF (Waters Ltd.) in negative electrospray mode with a scan range of 50–1000 m/z, capillary voltage 2·5KV, sampling cone 40V, desolvation temperature 450°C, source temperature 120°C and desolvation gas flow 800 L/h. Each analyte was identified according to its mass and retention time. Standard curves were performed, for bile acids, medium-chain fatty acids and long-chain fatty acids from 1 mg/ml stock solutions diluted to concentration ranges between 0·0064–20 µg/ml, using known standards. SCFA were quantified using GC-MS as previously described^(54,55)^. Bile acids and FA metabolites were quantified according to the standard curve and normalised according to the deuterated internal standards. MassLynx and TargetLynx applications by Waters were applied to sample data processing, quality and quantity determinations. Peaks for SCFA were integrated by using the Varian Star Chromatography Workstation version 6.0 software. Standards were included in each run to maintain calibration.

### Inflammatory and metabolic marker quantification

For measurement of adipokines (leptin and adiponectin), growth-arrest-specific 6, TNF*α*, IL-6, C-reactive protein and fasting insulin, venous blood was drawn into 2·5 ml vacuette tube (red top) which contained a serum separator clot activator (Grenier Bio-One International). Serum was allowed to clot at room temperature for approximately 30 min. Subsequently, the serum was separated by centrifugation (5000 ***g*** for 15 min). Serum concentrations for each inflammatory marker were determined using an ELISA (Protein Simple-Simple Plex Cartridge Kit, BioTechne). Samples were prepared and loaded into the cartridge according to a standard procedure provided by the manufacturers with all steps in the immunoassay procedure automated by the Ella instrument (Biotechne). All ranges for detection and quantification are provided in detail for each of the proteins evaluated from the company website documentation. For the measurement of fasting glucose venus blood was collected in an EDTA-treated collection tube (Sarstedt). For the measurement of HbA1c, TAG, total cholesterol, HDL, LDL, aspartate aminotransferase and alanine aminotransferase, venous blood was collected into a 2·5 ml vacuette tube (brown top), which contained a serum separator clot activator (Sarstedt). All markers of lipid/glucose metabolism and liver function (aspartate aminotransferase and alanine aminotransferase) were analysed by the Health Service Executive of Ireland in the biochemistry lab at University Hospital Limerick.

### Biostatistical analysis

All biostatistical analysis was carried out in Rstudio (version 4.1.1)^(57)^. The primary outcome measure (microbiota composition and diversity, measured using both *α* (*α*) diversity and beta (ß) diversity (Bray–Curtis dissimilarity and Weighted Unifrac). These measures were calculated using MetaPhlAn4 (version 4.05)^(52)^. To test for differences in ß-diversity between the groups, we used permutational analysis of variance. Principal component analysis was used to visualise differences in microbiota composition and conducted using the dudi.pco and s.class functions of the ade4 (version 1.7) package^(58)^. In order to establish significantly differentially abundant genera between the treatment and placebo groups over time we used Analysis of Compositions of Microbiomes with Bias Correction 2 (ANCOMBC2) (version 3.17). Differences in overall habitual diet (daily frequency of consumption), overall drug consumption and physical activity were visualised on a principal component analysis using a Kendall tau distances and tested using a permutational analysis of variance. In order to test for the differential consumption of specific food items between the treatment and placebo groups we used generalised linear mixed effects models examining the interaction between treatment and time as fixed effect and controlling for the patient identifier as a random effect. Logistic regression was used to determine whether any specific drugs were differentially consumed between the placebo and treatment groups at baseline. Generalised linear mixed effects models (using same approach for diet) were used to determine significance for fecal bile acid and fatty acid levels (secondary outcome measure) between the treatment and placebo groups. Statistical significance of other secondary outcome measures of this study, including markers of glycaemic control, inflammation and SCFA were all calculated using were calculated by employing a two-way mixed ANOVA controlling for the patient identifier as a random effect. Furthermore, Fishers exact test was used to determine significance between the number of patients with glycaemic control versus those without. All *P* values presented in this study were corrected for false discovery rate (FDR) using the Benjamini Hochberg method. Statistical significance for FDR values was determined using a cut-off of < 0·1, unless otherwise stated.

## Results

### Patient characteristics of treatment and placebo groups

We performed a randomised, placebo controlled, two-arm dietary fibre (yeast *β*-glucan) intervention in patients with T2DM. In total, thirty-nine individuals were recruited to the study with twenty-one being administered yeast *β*-glucan and eighteen being administered the placebo maltodextrin (Table 1 and Fig. 1). Three subjects withdrew from the intervention after 4 weeks (*n* = 1 from placebo group and *n* = 2 from the yeast *β*-glucan treatment group) (Fig. 1). Additionally, a total of three patients failed to produce a stool sample at any given time (*n* = 2 from the placebo and *n* = 1 from yeast *β*-glucan treatment group) (Fig. 1). See Table 1 for the complete overview of sample sizes and patient characteristics for both study groups. Patients in the yeast *β*-glucan treatment group had a similar age range (60 (sd 9·1) years) to patients administered maltodextrin (58·6 (sd 11·3) years). A similar gender distribution was observed among both groups (Table 1). Patients assigned to the placebo group consumed 14·5 g/d of dietary fibre habitually while those in the yeast *β*-glucan treatment group typically consumed 17 g/d.

**Table 1.** Subject charactersitics and sample sizes

	Placebo		Treatment	
	(Maltodextrin)		(Yeast *β*-glucan)	
Number of subjects	18		21	
Number of fecal samples				
Baseline	16		19	
4 weeks	16		18	
8 weeks	15		16	
Number of serum samples				
Baseline	18		21	
4 weeks	18		21	
8 weeks	15		18	
	*n*	%	*n*	%
Gender (% male) (*n*)	61·1	11	52·3	11
	Mean	sd	Mean	sd
Age (years) (Mean (sd))	58·6	11·3	60	9·1
Habitual fibre intake (g/d)	14·5		17	
	*n*	%	*n*	%
Smoking				
Non (%) (*n*)	55·5	10	57·2	12
Current (%) (*n*)	0	0	19	4
Ex (%) (*n*)	44·4	8	23·8	5
Alcohol				
> 2 units/d (%) (*n*)	38·0	7	23·8	5
< 2 units/d (%) (*n*)	61·1	11	76·2	16
Metformin (%) (*n*)	50·0	9	57·1	12
Statins (%) (*n*)	61·1	11	71·4	15
ACE inhibitor (%) (*n*)	55·5	10	14·2	3
Angiotensin 2 blocker (%) (*n*)	22·2	4	42·8	9
Aspirin (%) (*n*)	22·2	4	33·3	7
Proton pump inhibitor (%) (n)	33·3	6	28·5	6
Analgesic (%) (*n*)	33·3	6	28·5	6
SGLT2 inhibitor (%) (*n*)	33·3	6	23·8	5
Beta blocker (%) (*n*)	27·7	5	23·8	5
Incretin mimetics (%) (*n*)	19·0	4	23·8	5

We hypothesised that the treatment effect of yeast *β*-glucan on gut microbiota composition or serum metabolic and inflammatory markers could only be determined after identifying and adjusting for potential confounders. We tested all clinical metadata available to identify factors which were significantly different between both groups from the baseline to the endpoint. Principal component analysis revealed no significant differences for overall drug consumption (PERMANOVA FDR-corrected *P* = 0·346), (online Supplementary Fig. 1). Metformin and statins had the highest consumption levels across both treatment groups followed by drugs used to treat hypertension (ACE inhibitors, aspirin and angiotensin 2 blockers) (Table 1). However, logistic regression revealed that no individual drug was differentially consumed between the treatment and placebo groups (Supplementary File 2). Given that drug type or dose did not change for any subject over the course of the intervention we only used baseline measures for this analysis. Likewise, no significant changes could be detected for physical activity levels (online Supplementary Fig. 2), overall habitual diet (online Supplementary Fig. 3) or the consumption of individual dietary ingredients (Supplementary File 3) with respect to both treatment and time. We could not detect any significant differences between the yeast *β*-glucan treatment group and the placebo for any clinical or demographic metadata collected.

### Yeast *β*-glucan lowers insulin resistance in patients with Type II diabetes mellitus compared with placebo, independent of fecal microbiota and short-chain fatty acids alterations

**Figure 2.**
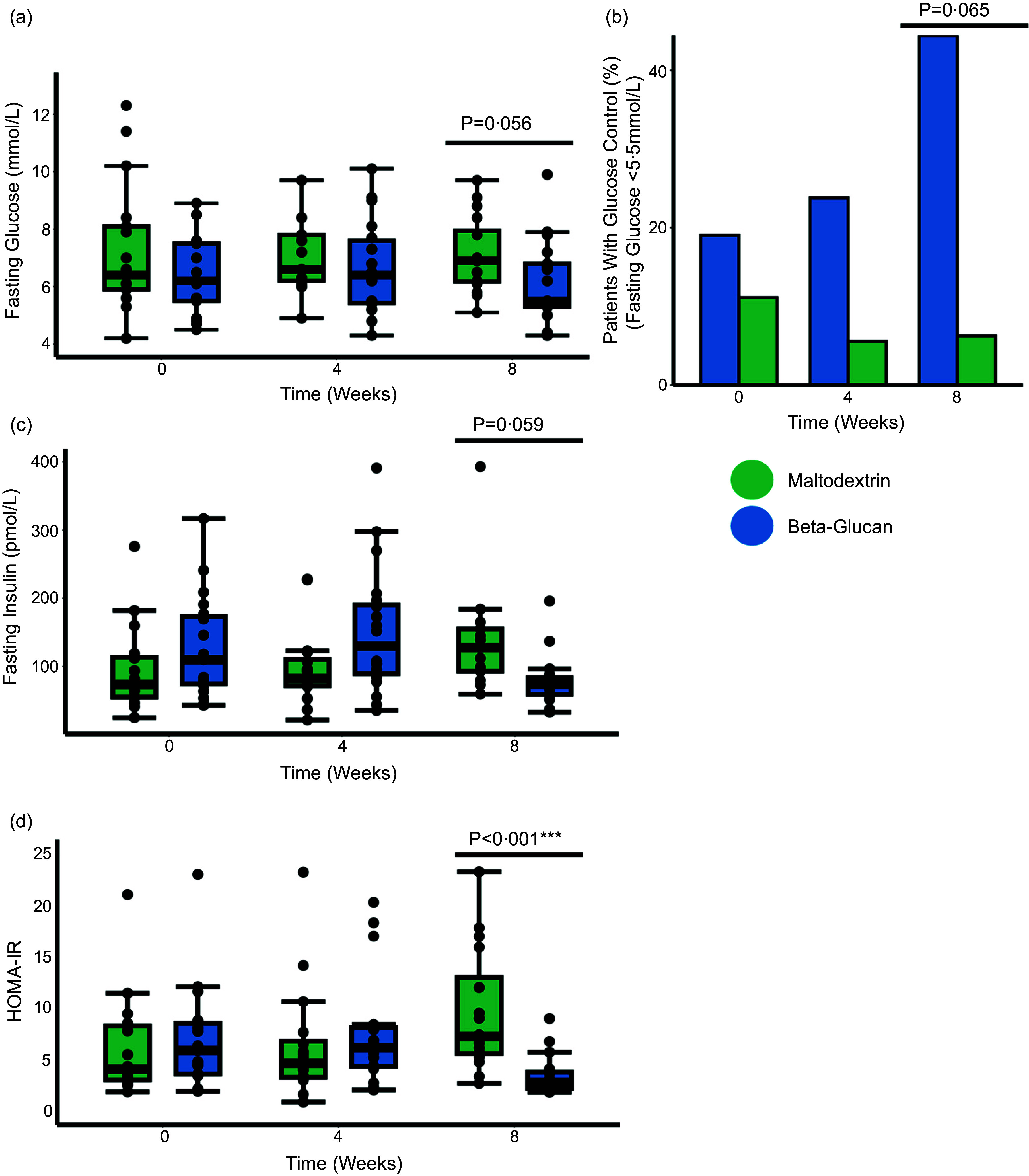
Yeast *β*-glucan improves glucose homeostasis in patients with T2DM independently of fecal microbiota alterations. (a) Boxplot of fasting glucose (mmol/l). (b) Boxplot of fasting insulin (pg/ml) and (c) boxplot of HOMA-IR (homeostatic model assessment for insulin resistance). All plots are colour coded with the yeast *β*-glucan represented as blue and the placebo maltodextrin represented as green. Statistical significance was determined for the boxplots using a two-way mixed ANOVA controlling for the patient identifier as a random effect. The annotations used for *P* values are *P* < 0·1 *; *P* < 0·05 **; *P* < 0·01 ***. All displayed *P* values are FDR corrected. FDR, false discovery rate.

The level of fasting glucose (mmol/l) (secondary outcome measure) remained unaltered in patients consuming yeast *β*-glucan when compared with the placebo maltodextrin after 8 weeks (FDR-corrected *P* = 0·056) (Fig. 2(a)). In addition, the percentage of patients in the yeast *β*-glucan group who had adequate glycaemic control (fasting glucose < 5·5 mmol/l) after 8 weeks was not significantly higher than the placebo group (FDR-corrected *P* = 0·065) (Fig. 2(b)). Similar findings were observed for fasting insulin levels at the study endpoint (FDR-corrected *P* = 0·059) (Fig. 2(c)). Furthermore, we found that HbA1c levels remained unchanged over the course of the study regardless of treatment (online Supplementary Fig. 4(a) and (b)). Using both fasting glucose and insulin values, we calculated the homeostatic model assessment for insulin resistance (HOMA-IR), an estimation of insulin resistance. We found that patients administered yeast *β*-glucan had significantly lower insulin resistance levels at 8 weeks when compared with the placebo group (Fig. 2(d)). Importantly, for all measures of glycaemic control, no significant difference could be detected in the yeast *β*-glucan group over time (Fig. 2(a)).

Given that T2DM is characterised by dysfunctional lipid metabolism, we also measured TAG and cholesterol in serum. Interestingly, the majority of patients enrolled in this study had relatively normal baseline levels for TAG (< 1·7 mmol/l), LDL (< 2·6 mmol/l), HDL (> 1·2 mmol/l) and total cholesterol (< 5·2 mmol/l) (online Supplementary Fig. 5(a)–(d)) all of which remained relatively stable over the study period for both groups. We report that yeast *β*-glucan did not significantly alter serum lipid profiles. Furthermore, we could not detect any changes to anthropometric measurements (weight and BMI) in response to the yeast *β*-glucan intervention.

Principal component analysis of species level *β*-diversity (Fig. 3(a)–(e) and online Supplementary Fig. 6–8) and ANCOMBC differential abundance analysis (mixed effect modelling) (online Supplementary File 4) revealed that yeast *β*-glucan did not significantly alter microbiota composition in patients with T2DM. Thus, no significant changes could be detected for the primary outcome measure of this study. In addition, we were also unable to detect changes to fecal SCFA levels (Supplementary File 5). Thus, our findings indicate that yeast *β*-glucan can lower insulin resistance in patients with T2DM independently of this mechanism.

**Figuew 3.**
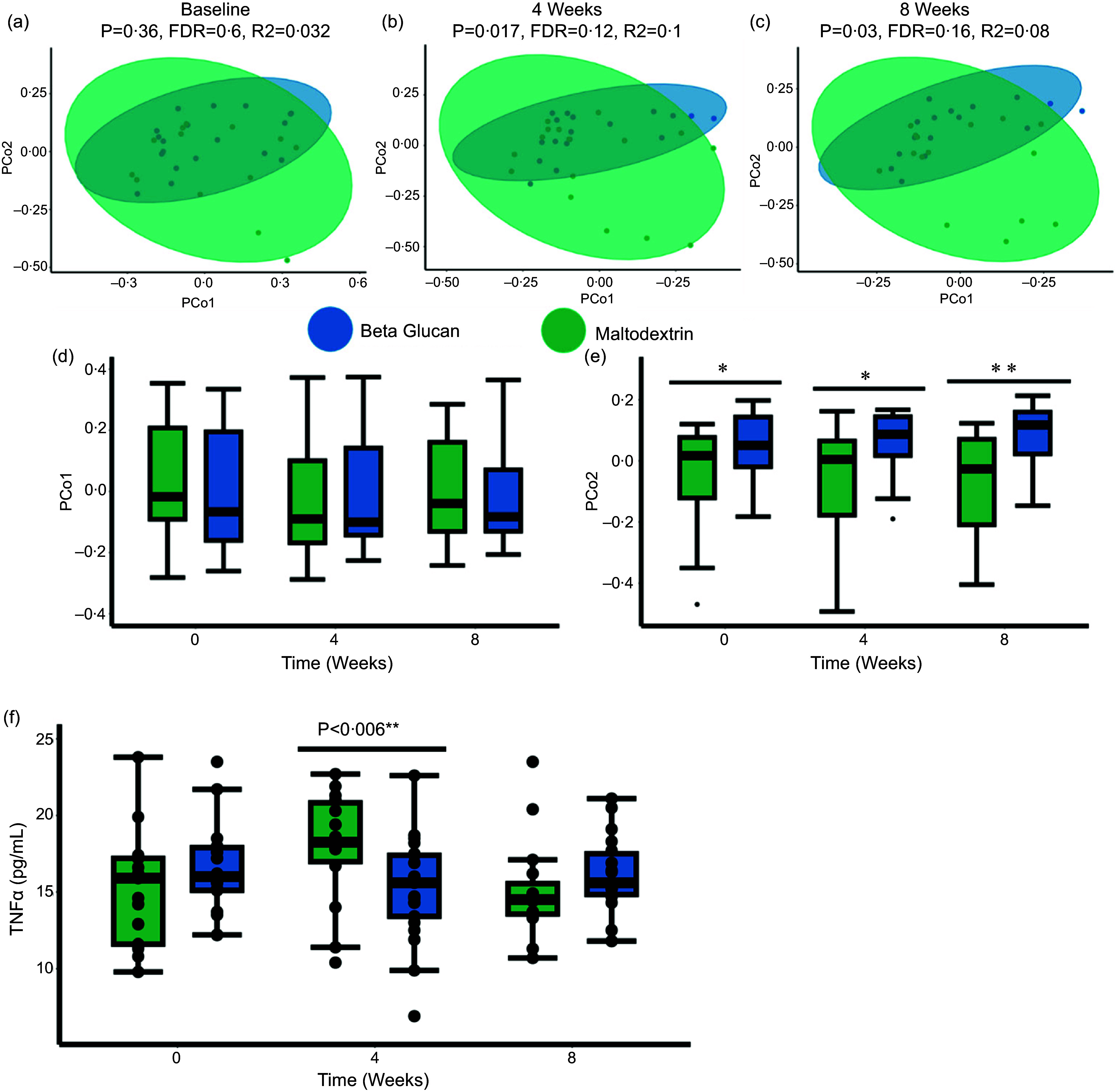
Yeast *β*-glucan does not significantly alter microbiota composition compared with the placebo maltodextrin. Principal component analysis (PCoA) of *β*-diversity (Bray–Curtis dissimilarity) at the species level (shotgun metagenomic sequencing profiles) between the placebo maltodextrin and yeast *β*-glucan groups at (a) baseline, (b) 4 weeks and (c) 8 weeks. Coordinates of the PCo1 (d) and the PCo2 (e) axis are shown as a boxplot. (f) Boxplot of TNF*α* (pg/ml). Statistical significance was determined for the boxplots using a two-way mixed ANOVA controlling for the patient identifier as a random effect. All plots are colour coded with the yeast *β*-glucan represented as blue and the placebo maltodextrin represented as green. The annotations used for *P* values are *P* < 0·1 *; *P* < 0·05 **; *P* < 0·01***. All displayed *P* values are FDR corrected. FDR, false discovery rate.

**Figure 4.**
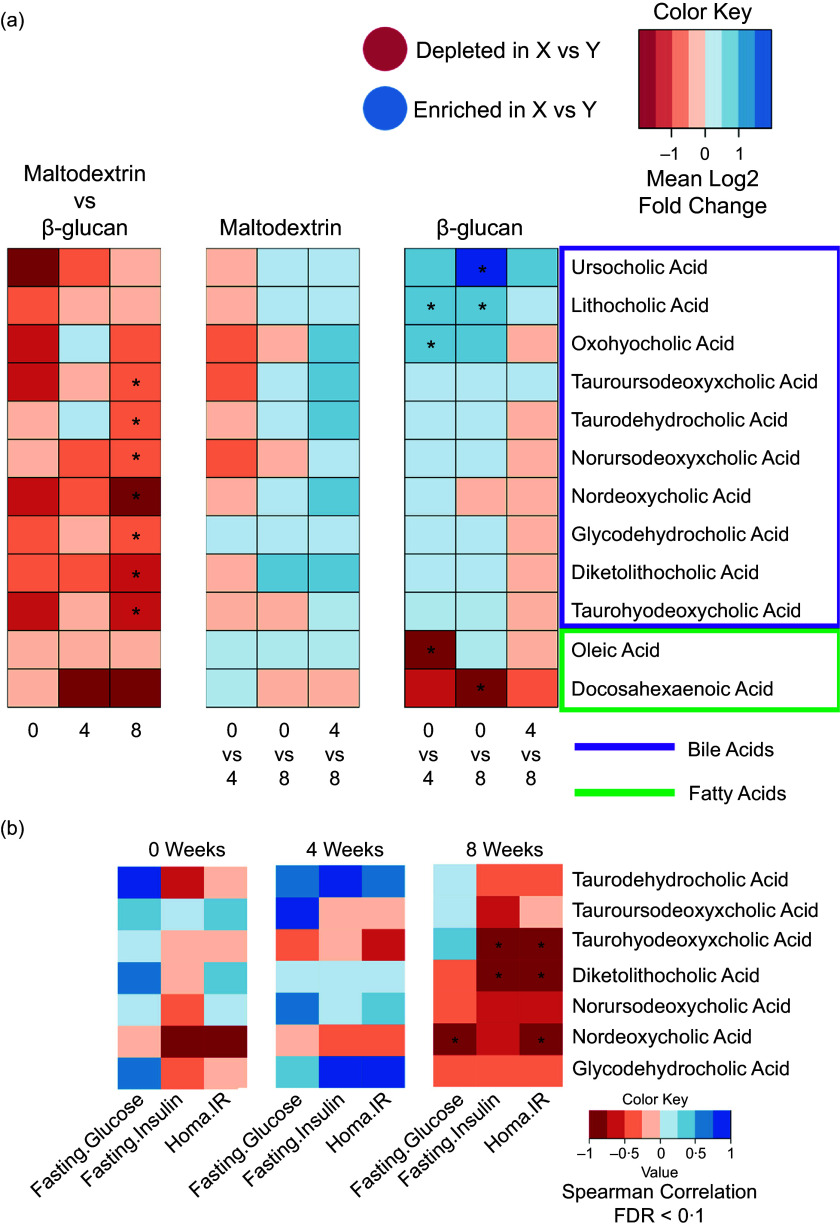
Precision bile acid and fatty acid alterations in response to yeast *β*-glucan supplementation. (a) Heatmap showing the mean log2 fold change between all pairwise comparisons for bile acid and fatty acid levels as determined using ultra-chromatography mass spectrometry relative to time (weeks). Fatty acids are highlighted in green, while bile acids are highlighted in purple. The directionality of each pairwise comparison is highlighted in the legend above the heatmap, whereby red depicts a metabolite depleted in X vs Y and blue depicts a metabolite that is enriched in X vs Y (i.e. maltodextrin (X) versus yeast *β*-glucan (Y) or 0 weeks (X) versus 4 weeks (Y). Significance was determined using GLMM, where the interaction between treatment and time was a fixed effect, and the patient identifier was controlled for as a random effect. Only metabolites that are significant are shown. (b) Heatmap showing spearman correlations between markers of glucose metabolism and bile acids identified as being significantly different from Fig. 3(a). All displayed *P* values are FDR corrected. The annotations used for *P* values are *P* < 0·05 *. FDR, false discovery rate.

### Yeast *β*-glucan lowers inflammation levels after 4 weeks

Dietary fibre has been widely described in the literature to have anti-inflammatory properties^(59–61)^. Given that T2DM is associated with low-grade systemic inflammation, we measured several serum markers of inflammation (TNF*α*, IL-6, C-reactive protein, growth-arrest specific 6, leptin and adiponectin) to test the anti-inflammatory effects of yeast *β*-glucan. We report that serum TNF*α* (pg/ml) was significantly lower in patients administered yeast *β*-glucan after 4 weeks compared with those consuming the placebo maltodextrin (Fig. 3(f)). However, at the end of the intervention (8 weeks), we could not detect any significant difference between the groups. IL-6, C-reactive protein, growth-arrest specific 6 and the adipokines (leptin and adiponectin) remained unchanged in response to yeast *β*-glucan throughout the intervention (Supplementary File 6). Overall, these findings highlight the potential anti-inflammatory effect of yeast *β*-glucan in treating low-grade systemic inflammation, which is characteristic of T2DM.

### Bile acid alterations in response to yeast *β*-glucan supplementation associate with improved glycaemic control

We used targeted metabolomics to quantify bile acids and fatty acids from feces. For the full list of metabolites analysed through UPLC-MS see Supplementary File 7. Raw data used for all metabolomics analysis are available in Supplementary File 8. We detected that several BAs were significantly different between the yeast *β*-glucan and placebo groups (Fig. 3(a)). We employed generalised linear mixed-effects modelling, testing for the interaction between treatment and time as fixed effects, while accounting for the patient identifier as a random effect. Using this approach, we show that seven BA were significantly higher in patients administered yeast *β*-glucan than those in the placebo group after 8 weeks (Fig. 3(a) and Supplementary File 9). This included several conjugated BA such as tauroursodeoxycholic acid, taurohyodeoxycholic acid, taurodehydrocholic acid and glycodehydrocholic acid. In addition, we also found nordeoxycholic acid, norursodeoxycholic acid and 6,7-diketolithocholic acid to be significantly higher in the yeast *β*-glucan group (Fig. 3(a) and online Supplementary File 9). We also report that 7-oxohyocholic acid and lithocholic acid were significantly lower in patients administered yeast *β*-glucan after 4 weeks when compared with their own baseline levels (Fig. 3(a)). Lithocholic acid and ursocholic acid remained significantly lower after 8 weeks when comparing the yeast *β*-glucan group to their own baseline. We also found oleic acid and docosahexaenoic acid were significantly higher at 4 and 8 weeks, respectively, in patients consuming yeast *β*-glucan (as compared with their own baseline). Next, we wanted to establish whether the fecal bile acid levels associated with markers of glucose metabolism. In order to do so, we conducted spearman correlations between fasting glucose, fasting insulin and HOMA-IR with the seven bile acids identified as being significant in Fig. 3(a). Using this approach, we found several strong negative correlations between both sets of markers at week 8 (Fig. 3(b)). However, no significant associations were detected at any other time point. Both fasting glucose levels and HOMA-IR had strong negative correlations with the bile acid nordeoxycholic acid. Furthermore, HOMA-IR and fasting insulin were also found to negatively associate with 6,7-diketolithocholic acid and taurohyodeoxycholic acid.

## Discussion

Previously, it was reported that yeast *β*-glucan (Wellmune) supplementation ameliorated hyperinsulinaemia and insulin resistance in a pre-clinical murine model^(62)^. The current study builds on this finding as we report that yeast *β*-glucan consumption for 8 weeks in patients with T2DM improves lowers insulin resistance when compared with a placebo group. HOMA-IR (insulin resistance) was significantly lower in patients supplemented with yeast *β*-glucan compared with patients consuming maltodextrin placebo after 8 weeks. Interestingly, the reported effect of yeast *β*-glucan observed after 8 weeks coincided with the enrichment of several bile acids compared with the placebo. The differences detected between the placebo control and yeast *β*-glucan groups were independent of alterations to microbiota composition (the primary outcome measure) and fecal SCFA levels. Overall, the findings in this study highlight the potential of yeast *β*-glucan to be used a treatment to lower insulin resistance in patients with T2DM.

One of the key mechanisms proposed for the health promoting effect of dietary fibre is that higher consumption can selectively promote the abundance of colonic microbiota capable of fermentation, which is accompanied by increased production and availability of SCFA. Previously, it has been shown that *β*-glucans can significantly alter microbiota composition^(63–65)^ with significantly higher levels of microbiota encoding *β*-glucanase enzymes (capable of *β*-glucan fermentation) detected in response. This includes members of the *Clostridium* family, which are butyrate producers^(66–68)^. However, in this study, we report that fecal microbiota composition and SCFA levels remained unchanged over the course of the intervention in response to 2·5 g/day of yeast *β*-glucan. Dietary fibre- mediated microbiota and SCFA alterations have been shown to be dose dependent, which may in part explain why the microbiota remained unchanged^(69–72)^. In addition, the habitual fibre intake of participants included in this study was a mean 17 g/d. It is plausible that a dose of 2·5 g/d in addition to what was already being consumed was not large enough to detect a change in microbiota composition. With respect to this, we hypothesise that doses larger than 2·5 g/d of yeast *β*-glucan (Wellmune) are required to determine the effect of yeast *β*-glucan on fecal microbiota composition.

The structural composition of yeast *β*-glucan mean it is a soluble and viscous fibre^(73)^. It has been proposed that dietary fibres with such properties can modulate postprandial glycaemic response by lowering the rate of glucose diffusion through the unstirred water layer^(74–76)^. In addition, the increased viscosity of intestinal content is thought to reduce the capacity of *α*-amylase to convert starch to glucose^(74,76)^. In addition, it has been hypothesised that the viscous properties of *β*-glucan also allow it to sequester bile acids preventing their reabsorption in the terminal ileum^(77)^. Ultimately, this would increase the concentration of bile acids entering the colon for excretion through feces^(78–81)^. In the current study, we report that seven different bile acids (including TUDCA) were significantly higher in feces of patients supplemented with yeast *β*-glucan than those supplemented with maltodextrin after 8 weeks. Bile acids are potent signalling molecules capable of regulating metabolic and inflammatory function through the activation of nuclear and G-protein-coupled receptors^(82–87)^. Interestingly, TUDCA has been shown to positively regulate glucose homeostasis across multiple studies^(88–91)^. It has also been reported to regulate insulin signalling (PI3K/Akt pathway activation) through activation of sphingosine-1-phosphate receptor 2^(92)^. Meanwhile, TUDCA was also shown to lower insulin resistance by reducing ER stress^(93,94)^ and by increasing insulin degradation and clearance through increased expression of the insulin-degrading enzyme^(95)^.

Dietary fibre has also been reported to influence inflammation through interaction with surface receptors of epithelial and immune cells, independently of the microbiota^(96)^. In the current study, we reported that yeast *β*-glucan lowered serum TNF*α* after 4 weeks of supplementation when compared with the placebo control. Previously, an *in vitro* study showed that *β*-glucans significantly lowered TNF*α* levels in a RAW 264·7 macrophage cell line^(97)^. It has been suggested that *β*-glucan can the regulate inflammatory response through interactions with dendritic cells and activation of Toll-like receptors^(96,98,99)^.

One limitation of this study was the use of maltodextrin as a placebo control. Although historically, maltodextrin has been widely used as a placebo control ingredient in dietary fibre interventions, a recent meta-analysis by Almutairi *et al.* (2022) showed that maltodextrin had an effect on host physiology and gut microbiota composition in a large number of studies^(100)^. Although we did not detect maltodextrin as having an effect on any parameters measured, given the findings reported by Almutairi *et al.* (2022), future studies investigating the role of yeast *β*-glucan on human health should use an alternative placebo control product. A second limitation of this study was that both yeast *β*-glucan and the maltodextrin placebo were not identical products and thus this was not a blinded intervention. While several significant differences could be detected for markers of glycaemic control between the yeast *β*-glucan and maltodextrin groups, no significant differences could be detected in the yeast *β*-glucan over time. The limited sample size used in this current study aimed to determine significance in the primary outcome measure (microbiota composition). Future studies with a larger sample size, calculated specifically towards glycaemic control, are needed to further examine the effect of yeast *β*-glucan in T2DM.

The prevalence of T2DM continues to rise globally as it accounts for over one million deaths per year globally, making it one of the leading causes of mortality^(101)^. Despite advancements in medical research and clinical care the incidence of T2DM continues to rise. This current study highlights the potential of yeast *β*-glucan (Wellmune) in lowering insulin resistance in T2DM, which could help mitigate the burden of this disease on healthcare systems across the globe. Further investigations will need to be carried out in order to better understand the dose–response effect of yeast *β*-glucan (Wellmune), enabling the optimisation of dosage efficacy.

## Supporting information

Cronin et al. supplementary material 1 Cronin et al. supplementary material

Cronin et al. supplementary material 2 Cronin et al. supplementary material

Cronin et al. supplementary material 3 Cronin et al. supplementary material

Cronin et al. supplementary material 4 Cronin et al. supplementary material

Cronin et al. supplementary material 5 Cronin et al. supplementary material

Cronin et al. supplementary material 6 Cronin et al. supplementary material

Cronin et al. supplementary material 7 Cronin et al. supplementary material

Cronin et al. supplementary material 8 Cronin et al. supplementary material

Cronin et al. supplementary material 9 Cronin et al. supplementary material

Cronin et al. supplementary material 10 Cronin et al. supplementary material

## Supplementary material

For supplementary material/s referred to in this article, please visit https://doi.org/10.1017/S0007114524002526
