# Supplementary material for: Yeast β-glucan supplementation lowers insulin resistance without altering microbiota composition compared with placebo in subjects with type II diabetes: a phase I exploratory study
Source: Br J Nutr. 2024 Oct 23;132(9):1161–72. doi: 10.1017/S0007114524002526 (PMC11617109; doi:10.1017/S0007114524002526)
Supplement: Cronin et al. supplementary material 1 — Cronin et al. supplementary material [file S0007114524002526sup001.docx]

**
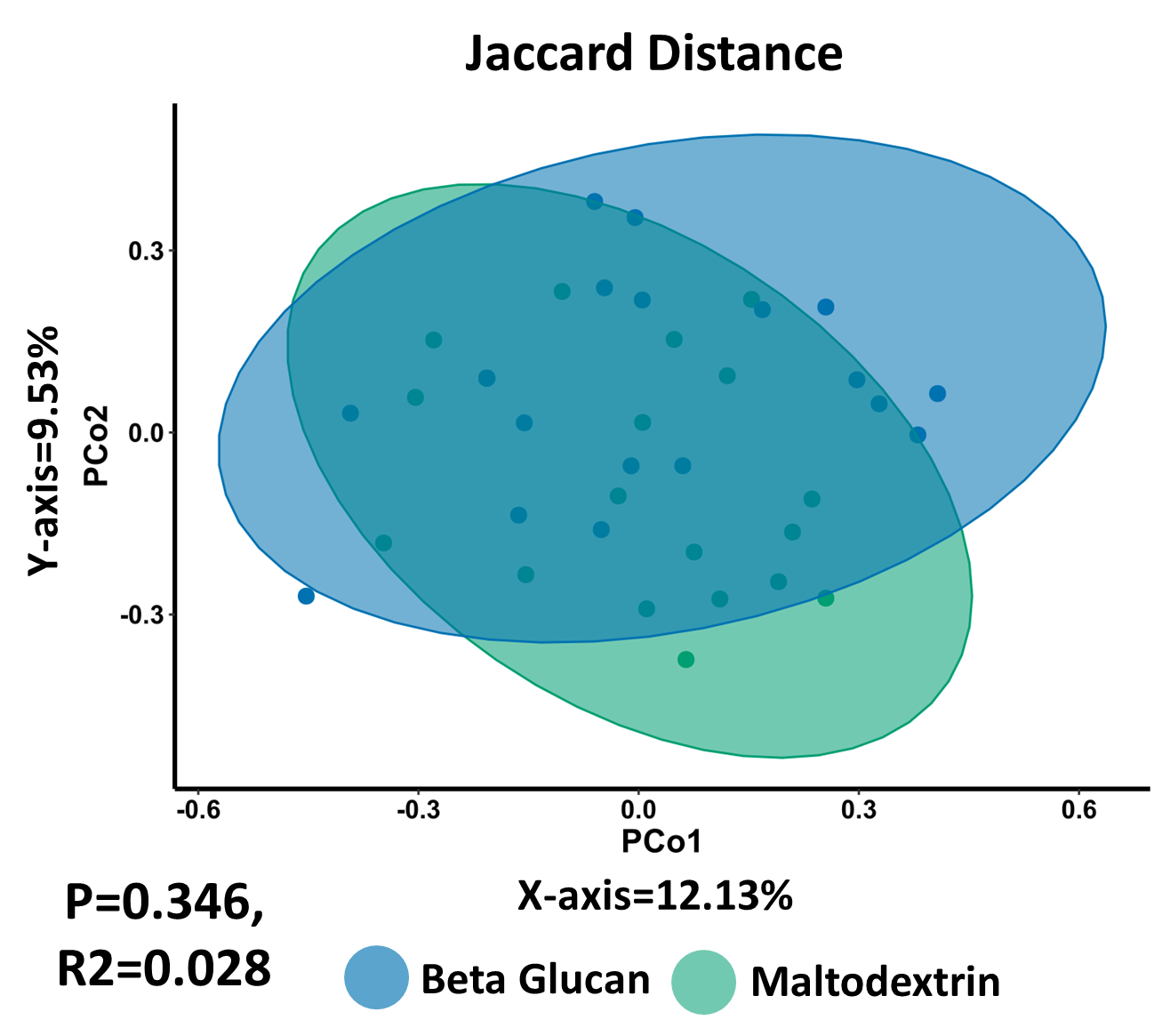
**

**Supplementary Figure 1:** No significant difference in overall drug consumption between the placebo and yeast 𝛽-glucan treatment groups. Principal Component Analysis (PCoA) of drug consumption profiles (Jaccard distance). The P Value (0.346) obtained using a PERMONOVA shows there is no statistically significant separation between the groups. The eigen values are also reported which show the variation reported in the X-axis (12.13%) and Y-axis (9.53%) of the PCoA.

**
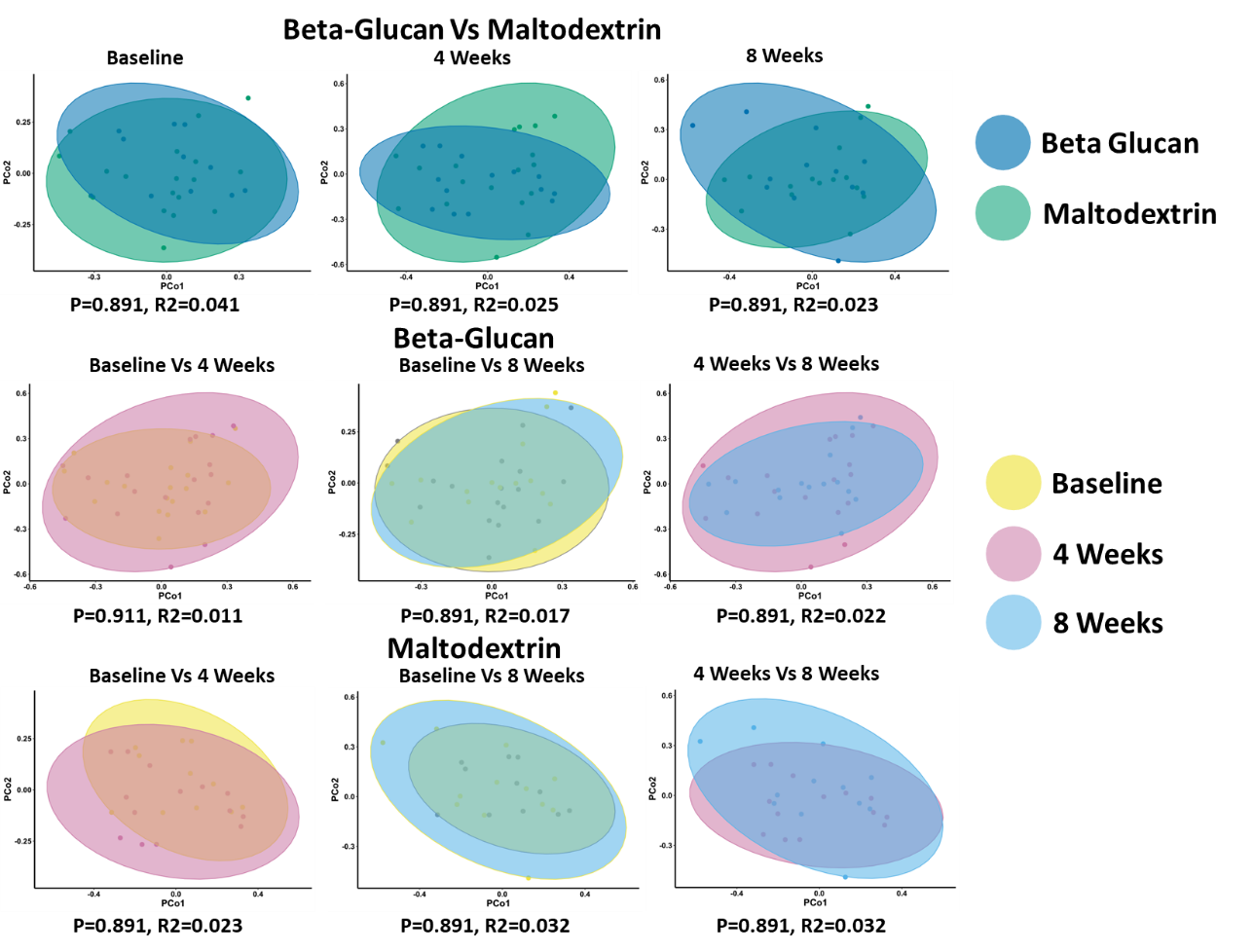
**

**Supplementary Figure 2:** No significant difference in physical activity levels between the placebo maltodextrin and yeast 𝛽-glucan treatment groups over time. Principal Component Analysis (PCoA) of physical activity profiles (Kendall tau distance). The P Values were obtained using a PERMONOVA shows there is no statistically significant separation with respect to both treatment and time. The eigen values are also reported which show the variation reported in the X-axis and Y-axis of the PCoA.

**
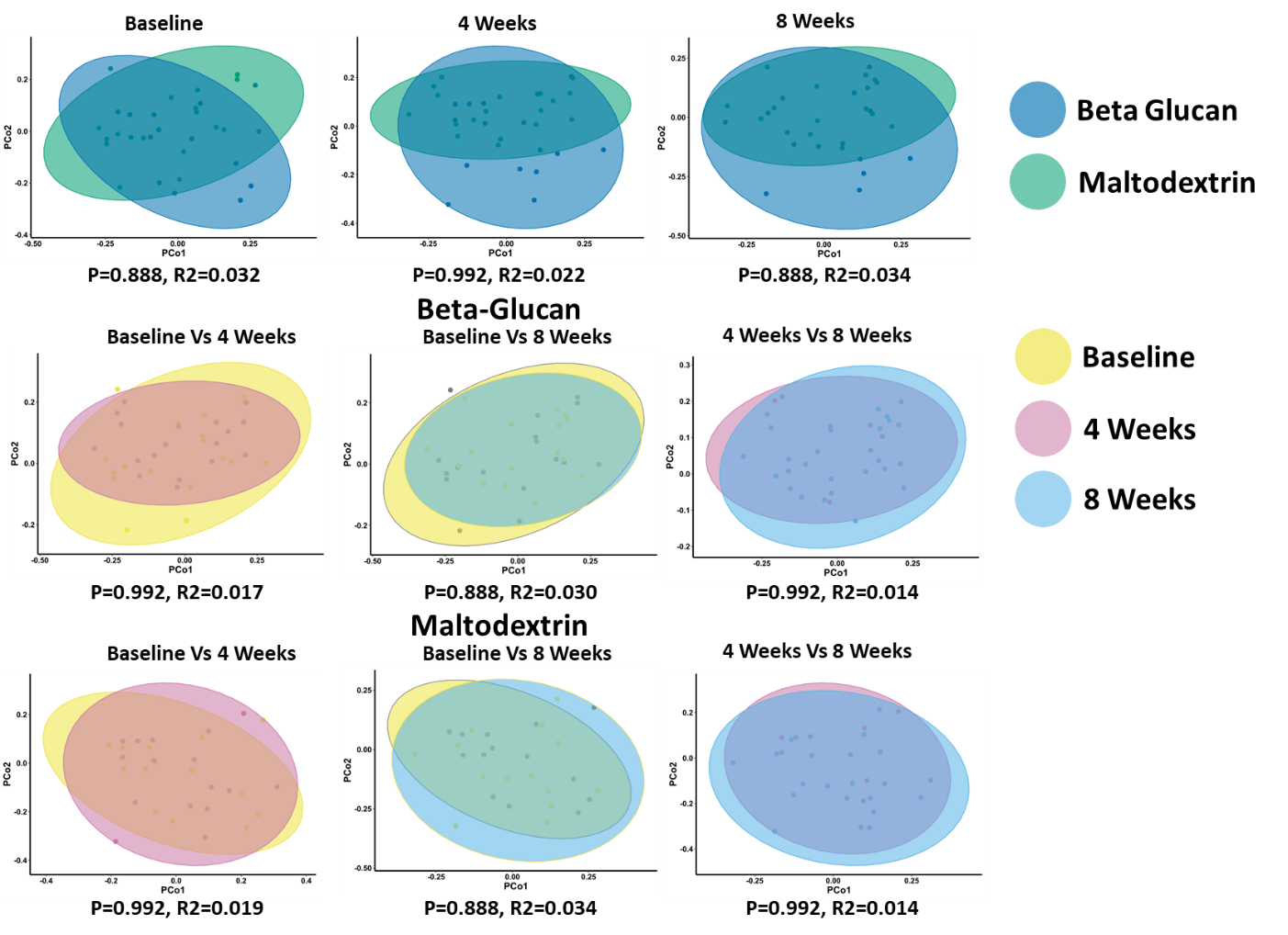
**

**Supplementary Figure 3:** No significant difference in habitual diet between the placebo maltodextrin and yeast 𝛽-glucan treatment groups over time. Principal Component Analysis (PCoA) of dietary profiles (Kendall tau distance) showing daily frequency of consumption. The P Values were obtained using a PERMONOVA shows there is no statistically significant separation with respect to both treatment and time. The eigen values are also reported which show the variation reported in the X-axis and Y-axis of the PCoA.


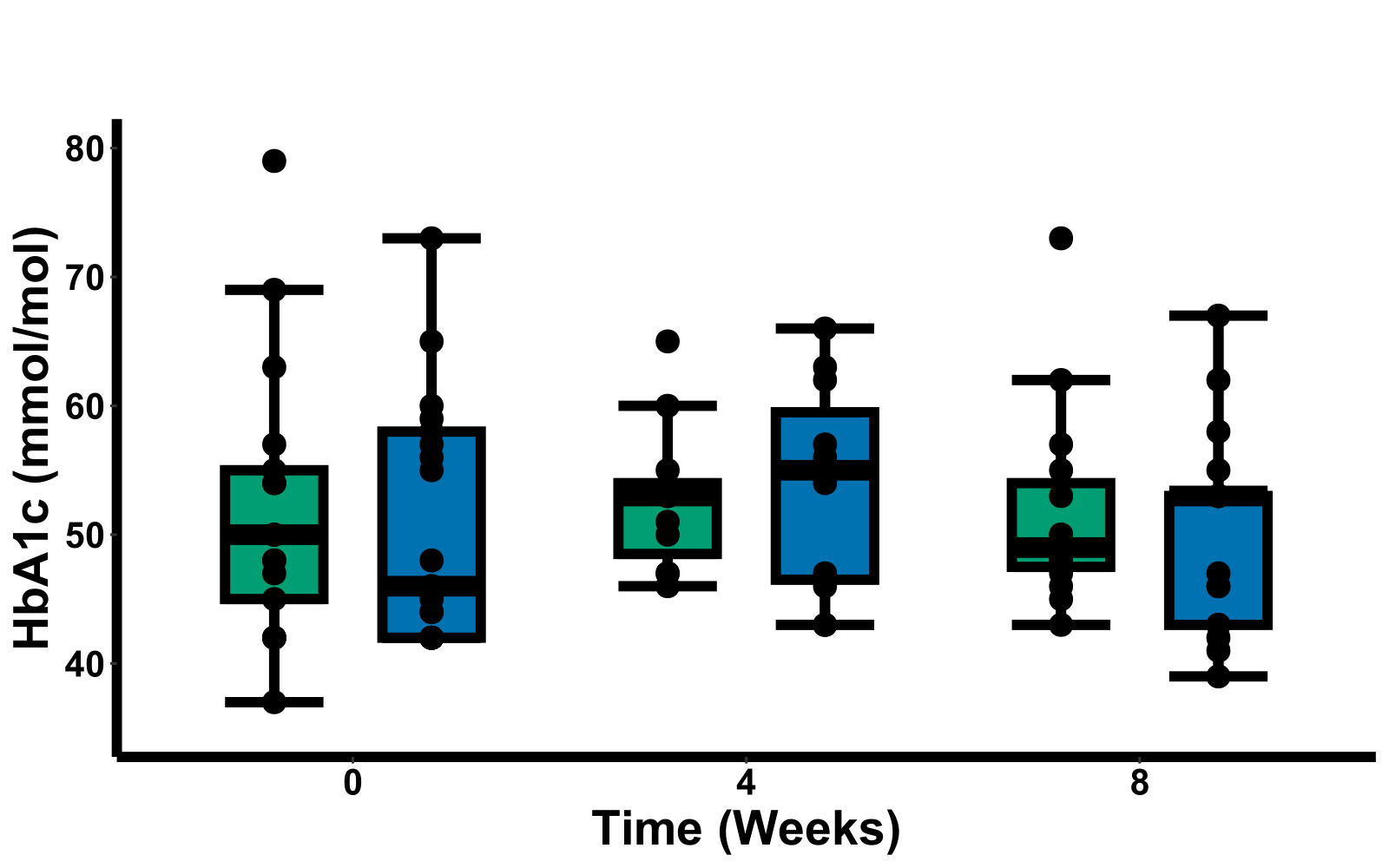


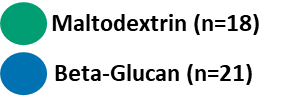


**
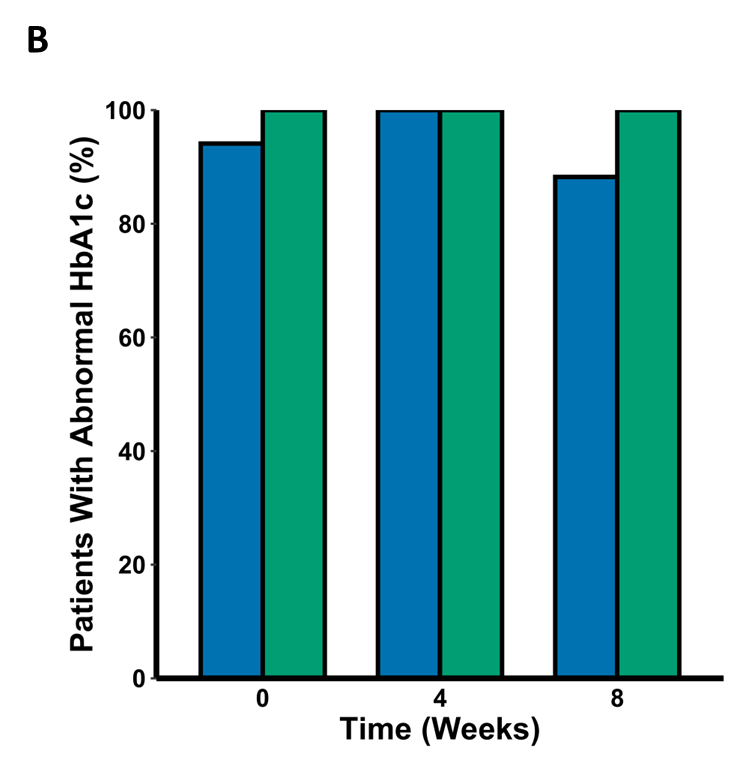
**

**Supplementary Figure 4:** No significant difference in plasma HbA1c between the placebo maltodextrin and yeast 𝛽-glucan treatment groups over time. A) Boxplot of HbA1c levels (mmol/mol) measured from plasma. Significance was tested using a two-way mixed ANOVA controlling for patient identifier as a random effect. B) Barplot showing the number of patients from each group observed to have abnormal HbA1c levels (< 48 mmol/mol). Significance for this measure was tested using Fishers exact test.


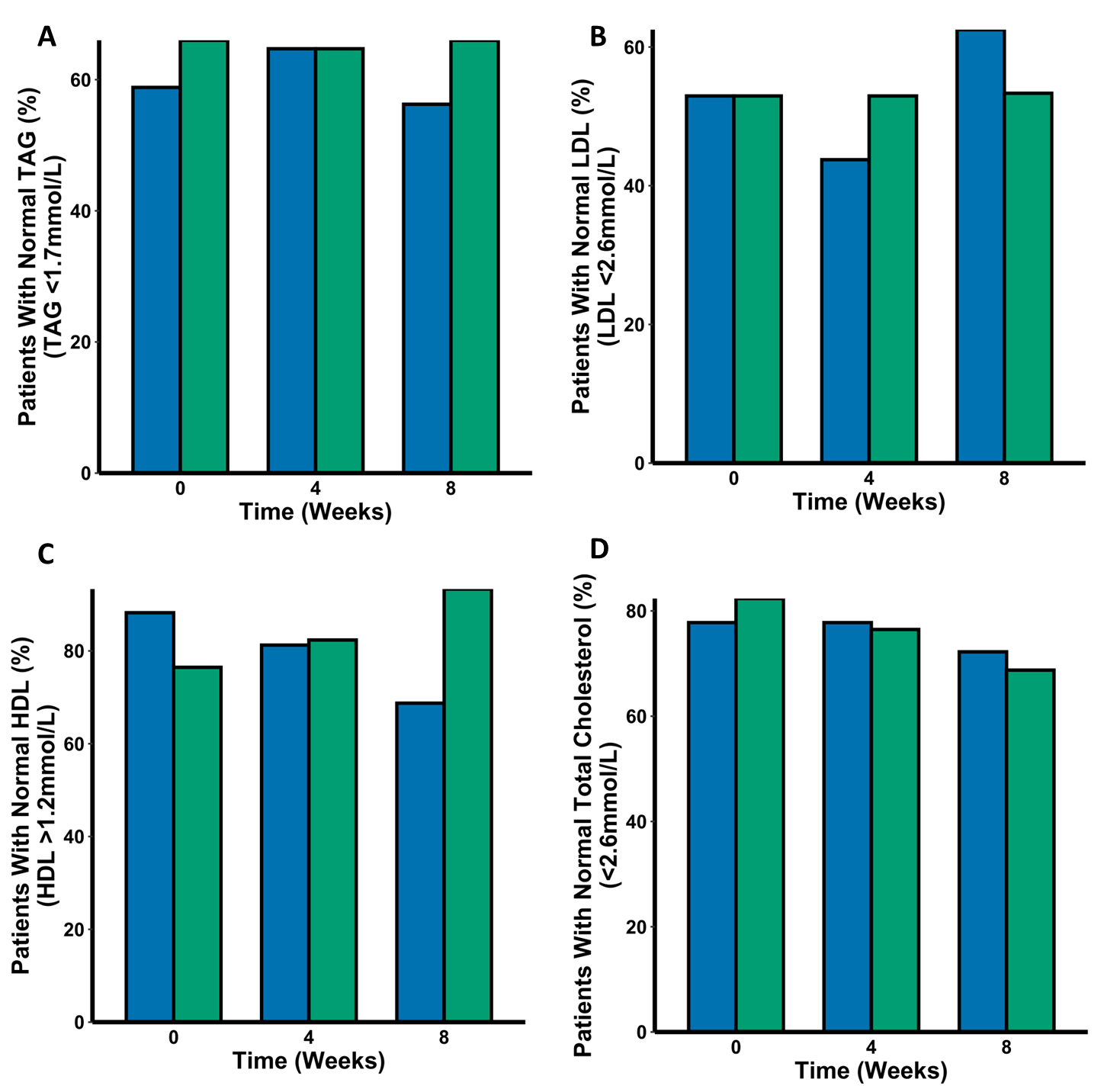


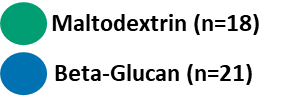


**Supplementary Figure 5:** No significant difference in the percentage of patients with normal serum lipid profiles between the placebo maltodextrin and yeast 𝛽-glucan treatment groups. Barplot of A) TAG, B) LDL, C) HDL and D) total cholesterol. Whether a patient was considered in the normal range for these lipids was decided as follows: TAG (<1.7mmol/L), LDL (<2.6mmol/L), HDL (>1.2mmol/L) and total cholesterol (<5.2mmol/L). Significance for this measure was tested using Fishers exact test.


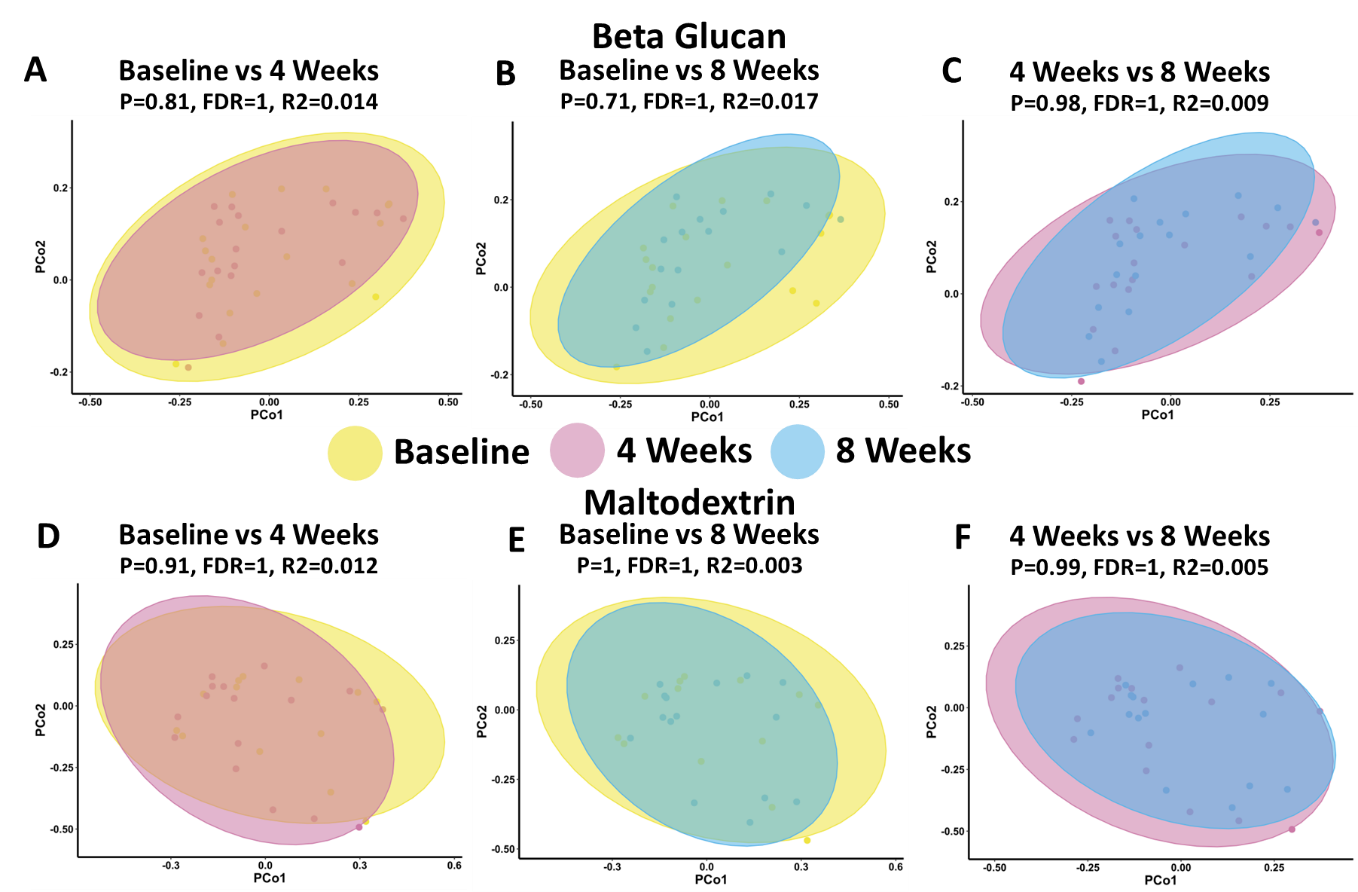


**Supplementary Figure 6:** Yeast 𝛽-glucan or maltodextrin does not significantly alter microbiota composition over time. Principal Component Analysis (PCoA) of β-diversity (Bray-Curtis dissimilarity) at the species level (shotgun metagenomic sequencing profiles) comparing differences in the yeast 𝛽-glucan group (A-C) or maltodextrin group (D-F) over time. All plots are colour coded with the yeast 𝛽-glucan represented as blue and the placebo maltodextrin represented as green.


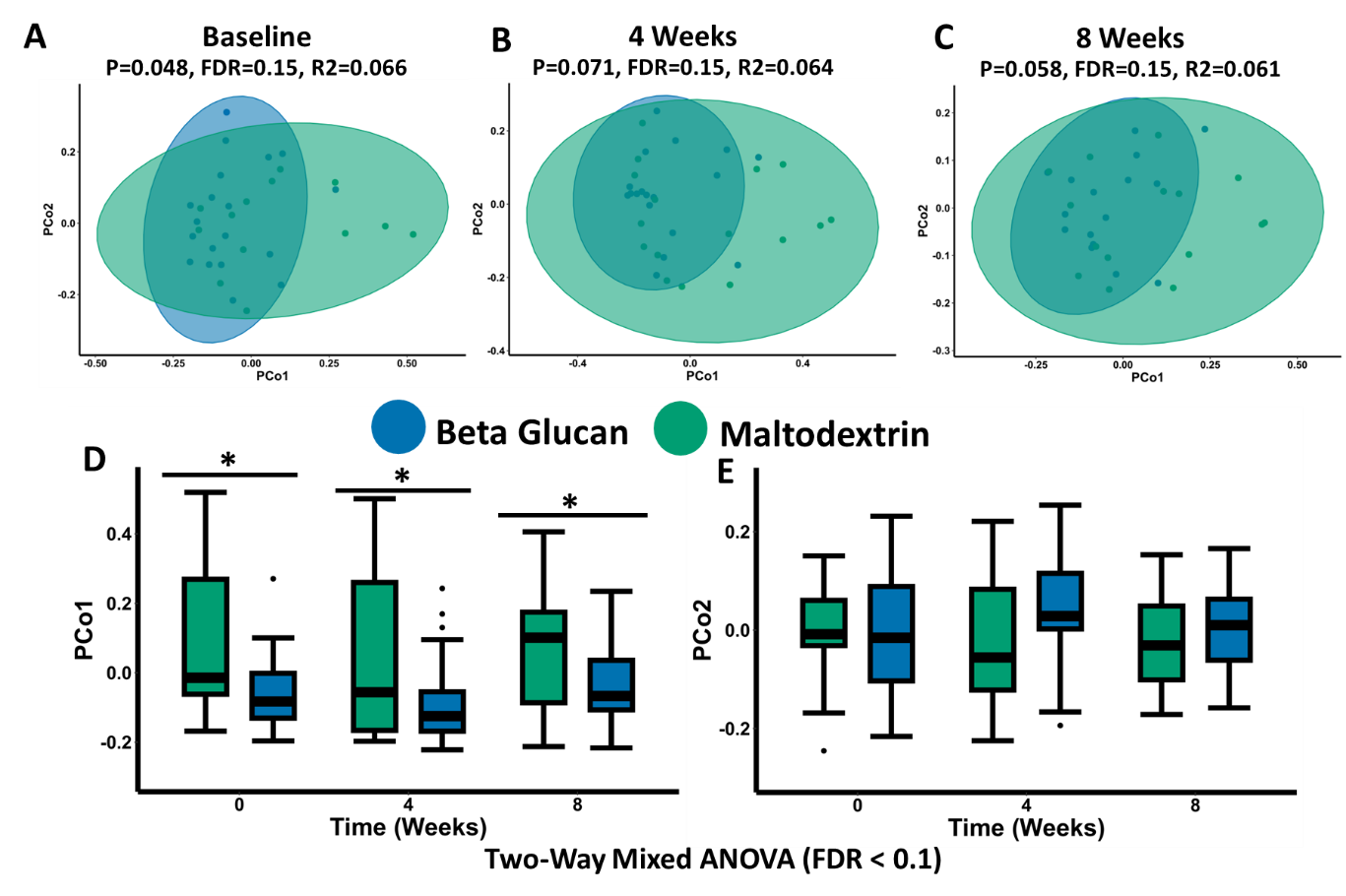


**Supplementary Figure 7:** Yeast 𝛽-glucan does not significantly alter microbiota composition compared to the placebo maltodextrin. Principal Component Analysis (PCoA) of β-diversity (Weighted Unifrac) at the species level (shotgun metagenomic sequencing profiles) between the placebo maltodextrin and yeast 𝛽-glucan groups at A) baseline, B) 4 weeks and C) 8 weeks. Coordinates of the PCo1 (D) and the PCo2 (E) axis are shown as a boxplot. Statistical significance was determined for the boxplots (PCo1 and PCo2 coordinates) using a two-way mixed ANOVA controlling for the patient identifier as a random effect. All plots are colour coded with the yeast 𝛽-glucan represented as blue and the placebo maltodextrin represented as green. The annotations used for P values are P < 0.1 *; P < 0.05 **; P < 0.01***. All displayed P values are FDR corrected.


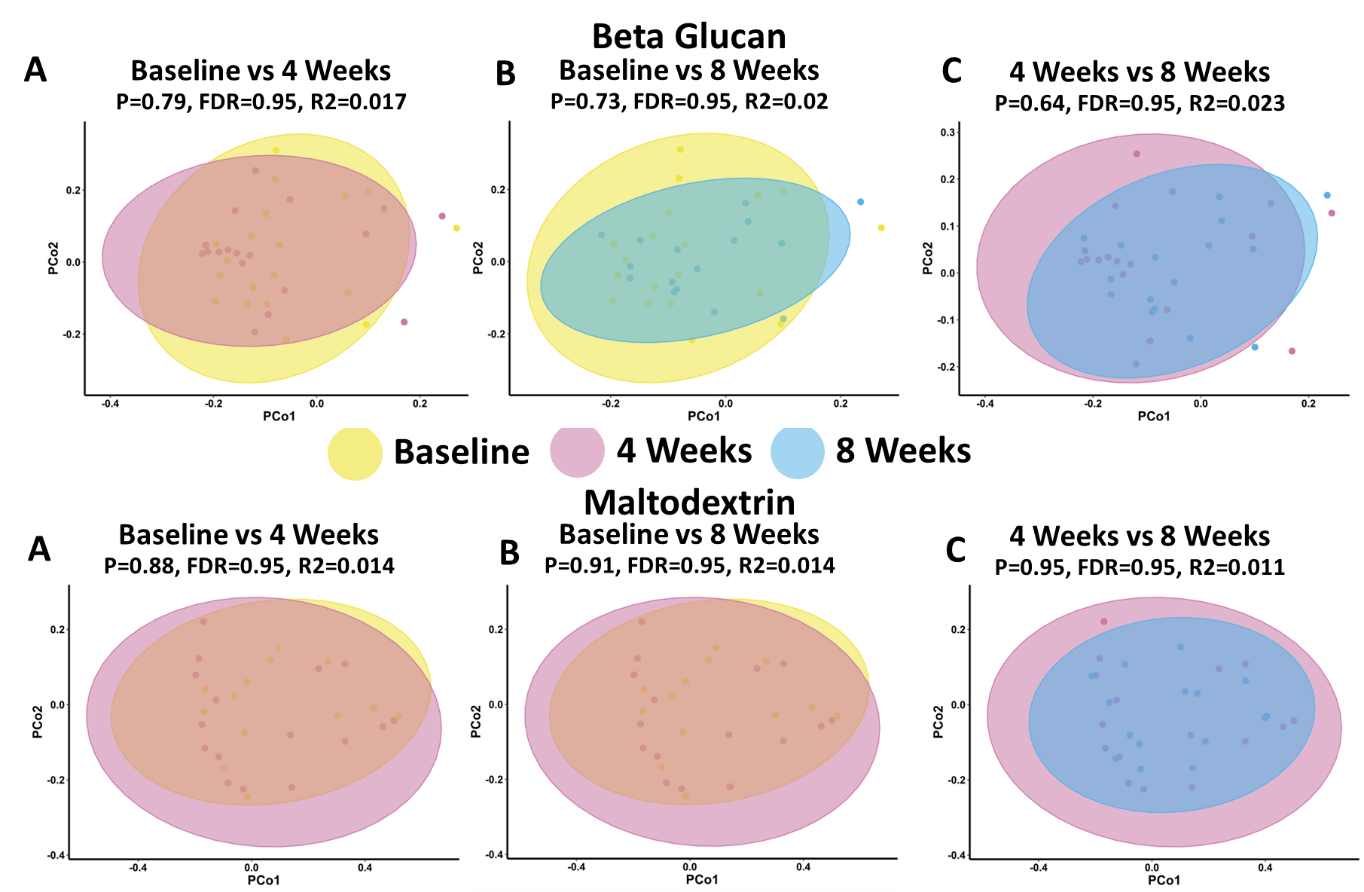


**Supplementary Figure 8:** Yeast 𝛽-glucan or maltodextrin does not significantly alter microbiota composition over time. Principal Component Analysis (PCoA) of β-diversity (Weighted Unifrac) at the species level (shotgun metagenomic sequencing profiles) comparing differences in the yeast 𝛽-glucan group (A-C) or maltodextrin group (D-F) over time. All plots are colour coded with the yeast 𝛽-glucan represented as blue and the placebo maltodextrin represented as green.
